# Stiff Person Syndrome: Bridging Neuroimmunology and Rehabilitation for Improved Patient Outcomes

**DOI:** 10.26502/aimr.0252

**Published:** 2026-07-28

**Authors:** Colleen C. Mercado, Marcel P. Fraix, Devendra K. Agrawal

**Affiliations:** 1 Departments of Translational Research and Physical Medicine and Rehabilitation.; 2 College of Osteopathic Medicine of the Pacific, Western University of Health Sciences, Pomona, California 91766 USA.

**Keywords:** Amphiphysin antibodies, Anti-glutamic acid decarboxylase (GAD65) antibody, Autoimmune disorder, Encephalomyelitis, Immunoglobulins, Inhibitory neurotransmission, Myoclonus, Neuroimmunology, Neurological disorder, Rehabilitation, Rituximab, Stiff Person Syndrome (SPS)

## Abstract

Stiff person syndrome (SPS) is a rare autoimmune neurologic disorder characterized by progressive muscle rigidity, painful stimulus-triggered spasms, and impaired inhibitory neurotransmission. Once considered a singular entity, SPS is now recognized as part of a broader spectrum of autoimmune neurologic disorders with diverse clinical phenotypes, antibody profiles, and functional outcomes. Although advances in neuroimmunology have improved understanding of disease mechanisms and expanded therapeutic options, the functional consequences of SPS and the role of rehabilitation remain comparatively underrepresented in the literature. This comprehensive review provides the current evidence regarding the epidemiology, immunopathogenesis, clinical spectrum, diagnosis, treatment, functional outcomes, and rehabilitation considerations of stiff person syndrome and stiff person spectrum disorders. A review of the literature was conducted using peer-reviewed publications identified through PubMed and Google Scholars. Priority was given to original research, clinical trials, systematic reviews, and landmark studies, with an emphasis on contemporary literature published within the past five years. Additional studies were included to provide historical context and highlight the evolution of current concepts. SPS is increasingly recognized as a heterogeneous autoimmune disorder associated with anti-glutamic acid decarboxylase, glycine receptor, and amphiphysin antibodies, including rituximab, hematopoietic stem cell transplantation, and chimeric antigen receptor T-cell therapy, offer promise for refractory disease. Despite advances in medical management, SPS continues to impose substantial functional limitations affecting mobility, activities of daily living, and quality of life. Rehabilitation interventions, including physical and occupational therapy, spasticity management, neuromodulation, and multidisciplinary care, demonstrate potential to improve functional outcomes, although evidence remains limited. SPS is a complex autoimmune disorder requiring multidisciplinary management that extends beyond immunotherapy alone. Continued advances in biomarker discovery, targeted immunotherapies, standardized functional outcome measures, and evidence-based rehabilitation strategies are essential to improve diagnosis, optimize functional recovery, and enhance long-term quality of life.

## Introduction

Stiff person syndrome (SPS) is a rare autoimmune neurologic disorder characterized by progressive muscle rigidity, painful spasms, and heightened sensitivity to external stimuli. First described by Frederick Moersch and Henry Woltman in 1956, SPS has evolved from a clinically defined condition into a complex spectrum of immune-mediated disorders involving impaired inhibitory neurotransmission within the central nervous system [1–4]. Subsequent discoveries, particularly the identification of autoantibodies against glutamic acid decarboxylase (GAD), established SPS as an autoimmune condition and provided a mechanistic link to disrupted gamma-aminobutyric acid (GABA)-mediated inhibition [5,6].

Over the past two decades, SPS has been increasingly recognized as part of a broader group of disorders collectively referred to as stiff person spectrum disorders (SPSD), which include variants such as stiff limb syndrome and progressive encephalomyelitis with rigidity and myoclonus (PERM). These conditions share overlapping clinical and immunologic features but vary widely in severity, antibody profiles, and prognosis [7–9]. The expanding recognition of glycine receptor and amphiphysin antibodies, in addition to anti-GAD antibodies, has further highlighted the heterogeneity of SPS and its underlying immune mechanisms [10,11].

Clinically, SPS presents with a combination of axial rigidity, stimulus-triggered spasms, and progressive functional impairment that can significantly impact mobility, independence, and quality of life [12,13]. Patients frequently experience delays in diagnosis due to the rarity of the condition and its overlap with various psychiatric, functional, and other neurologic disorders, including epilepsy and movement disorders [14,15]. In addition, SPS is often associated with other autoimmune conditions, particularly type 1 diabetes and thyroid disease, further supporting its classification as a systemic autoimmune disorder [16,17].

Despite advances in understanding its immunopathogenesis, the management of SPS continues to be challenging. Current treatment strategies mostly target both symptomatic relief and immune modulation, with benzodiazepines and baclofen commonly used for symptom control, and intravenous immunoglobulin (IVIG) representing the most evidence-supported immunotherapy [18–20]. However, treatment responses vary, and emerging therapies such as rituximab, hematopoietic stem cell transplantation, and chimeric antigen receptor T-cell (CAR-T) therapy are being explored for refractory disease [21–24].

Importantly, while much of the existing literature has focused on the immunologic and neurologic aspects of SPS, the functional consequences of the disease remain underemphasized. Patients often experience significant disability due to impaired mobility, falls, pain, and reduced ability to perform activities of daily living, highlighting the need for comprehensive, multidisciplinary care [25,26]. Rehabilitation interventions, including physical therapy, spasticity management, and neuromodulation, have shown potential benefits in improving functional outcomes, though evidence remains limited and largely derived from case reports and small series [27,28].

Given these gaps, a comprehensive understanding of SPS requires integration of immunologic, neurologic, and functional perspectives. This review aims to synthesize current evidence on the epidemiology, pathophysiology, clinical presentation, diagnostic evaluation, and management of SPS, with particular emphasis on rehabilitation considerations and functional outcomes relevant to physical medicine and rehabilitation practice.

### EPIDEMIOLOGY, RISK FACTORS, AND ASSOCIATED CONDITIONS

Stiff person syndrome (SPS) is a rare autoimmune neurologic disorder with a prevalence of approximately 1 to 2 cases per million individuals worldwide. Due to its rarity and frequent misdiagnosis, the true prevalence is likely underestimated [11,29]. SPS most commonly presents in adulthood, with a peak onset between the third and sixth decades of life, although cases have been reported in both pediatric and older populations [19,30]. A consistent epidemiologic feature of SPS is its female predominance, with reported female-to-male ratios ranging from approximately 2:1 to 3:1. This distribution mirrors that of many autoimmune diseases and supports an underlying immune-mediated etiology [12,31]. While SPS has been reported worldwide, large-scale epidemiologic data is limited, and most insights are derived from case series and retrospective cohort studies.

The primary risk factors for SPS are related to immune dysregulation and the presence of underlying autoimmune conditions. The strongest association is with anti-glutamic acid decarboxylase (GAD65) antibodies, which are present in most patients with classic SPS. High titers of these antibodies are associated with neurologic manifestations and may reflect a greater autoimmune response [6,9]. SPS is frequently associated with other autoimmune diseases, most notably type 1 diabetes mellitus, which is present in a significant proportion of patients. This association likely reflects shared immune mechanisms targeting GAD65, which is expressed in both pancreatic beta cells and the central nervous system [5,16]. Additional autoimmune conditions commonly reported in SPS include autoimmune thyroid disease, pernicious anemia, vitiligo, and celiac disease [17,32–34].

Genetic susceptibility is an emerging area of interest in SPS. Recent immunogenetic studies have identified potential associations with lymphocytic gene variants and immune regulatory pathways, suggesting that genetic predisposition may contribute to disease development in the setting of environmental or immunologic triggers [35]. Infectious triggers, such as viral illnesses, have also been proposed as potential initiating factors in susceptible individuals [36]. SPS is increasingly recognized as part of a broader spectrum of autoimmune and paraneoplastic disorders. In addition to its association with endocrine autoimmune diseases, SPS may occur in the context of malignancy, particularly in patients with amphiphysin antibodies. Paraneoplastic SPS has been linked to cancers such as breast cancer, lung cancer, and other solid tumors, and may present with more rapid progression or atypical features [37,38].

Beyond endocrine and oncologic associations, SPS has been reported in conjunction with other autoimmune neurologic disorders, including limbic encephalitis, cerebellar ataxia, and progressive encephalomyelitis with rigidity and myoclonus (PERM), reflecting its classification within GAD antibody-spectrum disorders [2,8,9]. These overlapping conditions further highlight the heterogeneity of SPS and its shared immunopathogenic mechanisms. Additional associations with systemic and rare conditions have been described, including latent autoimmune diabetes in adults (LADA), Hashimoto’s thyroiditis, and, more rarely, hematologic disorders such as polycythemia vera [39–43]. While these associations are often based on case reports, they reinforce the concept of SPS as a manifestation of broader immune dysregulation.

**Figure 1:**
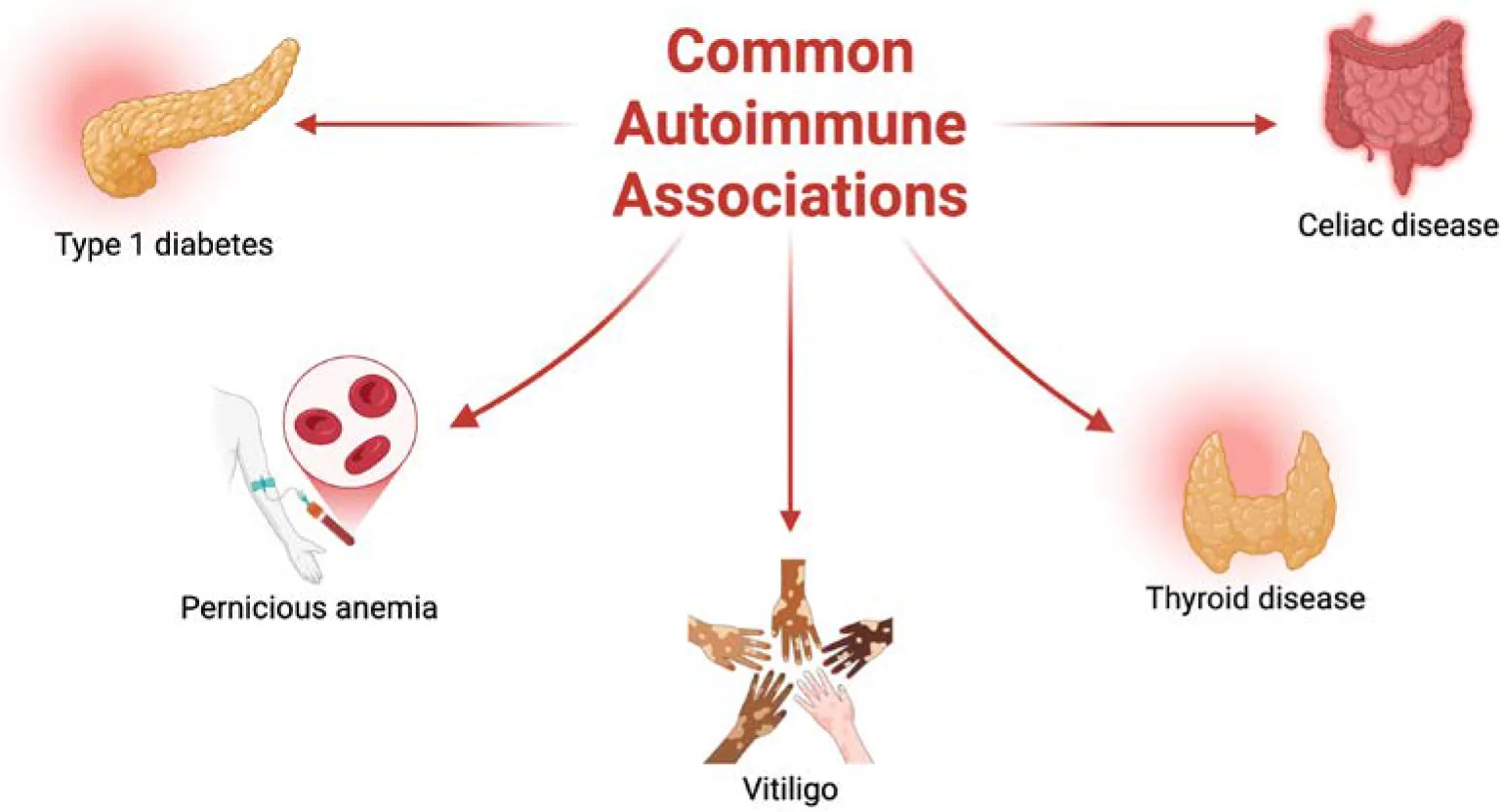
Common autoimmune associations in Stiff Person Spectrum Disorder.

Understanding the epidemiology and associated conditions of SPS has important clinical implications. The strong association with autoimmune diseases emphasizes the need for comprehensive screening in patients with SPS, including evaluation for endocrine and systemic autoimmune disorders (Figure 1). Similarly, recognition of paraneoplastic forms of SPS calls for appropriate malignancy screening, particularly in patients with atypical presentations or antibody profiles. Overall, SPS is best conceptualized as a rare but clinically significant autoimmune disorder with systemic associations and diverse presentations. Awareness of its epidemiologic features, risk factors, and associated conditions is essential for early diagnosis, appropriate evaluation, and comprehensive patient care.

### PATHOPHYSIOLOGY

Stiff person syndrome (SPS) is an autoimmune disorder characterized by impaired inhibitory neurotransmission within the central nervous system, leading to motor neuron hyperexcitability and the hallmark clinical features of rigidity and muscle spasms. The pathophysiology of SPS is primarily mediated by autoantibodies targeting proteins involved in gamma-aminobutyric acid (GABA)-ergic signaling, most notably glutamic acid decarboxylase 65 (GAD65), the rate-limiting enzyme responsible for GABA synthesis [5,44]. Anti-GAD65 antibodies are detected in most patients with classic SPS and are thought to decrease GABA production, resulting in reduced inhibitory signaling within spinal and supraspinal pathways. This loss of inhibitory tone leads to continuous motor unit activity and increased muscle stiffness, as demonstrated by characteristic electromyographic findings [6,45]. Evidence of intrathecal synthesis of anti-GAD antibodies further supports a central autoimmune process rather than a purely peripheral mechanism [46].

In addition to anti-GAD antibodies, other autoantibodies have been identified that contribute to the heterogeneity of SPS spectrum disorders. Glycine receptor (GlyR) antibodies are particularly associated with more severe phenotypes, including progressive encephalomyelitis with rigidity and myoclonus (PERM), and are thought to disrupt inhibitory neurotransmission at the level of the spinal cord and brainstem [7,10]. Experimental data suggest that GlyR antibodies impair presynaptic inhibitory signaling, further contributing to neuronal hyperexcitability and the development of severe motor and autonomic symptoms [47]. Additionally, amphiphysin antibodies are commonly associated with paraneoplastic SPS and may reflect an immune response triggered by underlying malignancy [37].

Recent advances have expanded the understanding of SPS into a broader category of GAD antibody-spectrum disorders, which include conditions such as autoimmune epilepsy, cerebellar ataxia, and limbic encephalitis. These disorders share common immunopathogenic mechanisms involving impaired inhibitory neurotransmission but manifest with diverse neurologic phenotypes depending on the regions of the central nervous system affected [8,9,11]. This spectrum-based framework highlights the importance of antibody specificity and distribution in determining clinical presentation.

At the cellular level, the loss of inhibitory neurotransmission leads to increased excitability of alpha motor neurons and reduced modulation of reflex pathways, resulting in sustained muscle contraction and exaggerated startle responses. Neurophysiological studies have demonstrated persistent motor unit firing and impaired reciprocal inhibition, supporting the concept of disinhibition as the central mechanism underlying SPS [45]. These findings correlate with the clinical observation of stimulus-triggered spasms and continuous muscle activity even at rest.

Emerging research has also begun to shed light on the genetic and immunologic factors contributing to disease susceptibility. Immunogenetic studies have identified potential associations with lymphocytic gene variants and immune regulatory pathways, suggesting the possibility that SPS may arise from a combination of genetic predisposition and environmental triggers [35]. Infectious triggers, such as viral illnesses, have also been proposed as potential initiating factors in susceptible individuals, further supporting the role of immune dysregulation in disease onset [36].

Overall, the pathophysiology of SPS reflects a complex interplay between autoantibody-mediated disruption of inhibitory neurotransmission, central nervous system hyperexcitability, and systemic immune dysfunction. The variability in antibody profiles and clinical manifestations underscores the heterogeneity of SPS spectrum disorders and has important implications for diagnosis, prognosis, and targeted therapeutic interventions.

### STIFF PERSON SPECTRUM DISORDERS

Stiff person syndrome (SPS) is now recognized as part of a broader group of immune-mediated neurologic conditions collectively referred to as stiff person spectrum disorders (SPSD). This spectrum includes a range of clinical phenotypes that share a common underlying mechanism of impaired inhibitory neurotransmission but differ in severity, distribution of symptoms, associated antibodies, and overall prognosis [8,9].

The concept of SPSD reflects the heterogeneity observed in patients with autoimmune disorders affecting gamma-aminobutyric acid (GABA)-ergic and glycinergic pathways. While classic SPS represents the classic presentation characterized by axial rigidity and stimulus-triggered spasms, other variants demonstrate more localized or more severe manifestations [7,31,48].

At the milder end of the spectrum, stiff limb syndrome is characterized by focal rigidity, typically involving a single limb, with less prominent axial involvement. In contrast, intermediate phenotypes may include SPS with additional autoimmune neurologic features, such as cerebellar ataxia or epilepsy, reflecting broader involvement of inhibitory circuits within the central nervous system [9]. At the severe end of the spectrum lies progressive encephalomyelitis with rigidity and myoclonus (PERM), a life-threatening condition characterized by widespread rigidity, brainstem involvement, myoclonus, autonomic instability, and encephalopathy. Patients with PERM often present with rapidly progressive symptoms and may require intensive care management [49,50].

The variability in clinical presentation across SPSD is closely linked to differences in underlying antibody profiles. Anti-glutamic acid decarboxylase (GAD65) antibodies are most associated with classic SPS and are typically linked to a more chronic, slowly progressive course [6,11]. In contrast, glycine receptor (GlyR) antibodies are more frequently associated with severe phenotypes, particularly PERM, and are thought to contribute to prominent brainstem and autonomic dysfunction [7,10]. Amphiphysin antibodies are typically associated with paraneoplastic SPS and may indicate an underlying malignancy, often accompanied by more rapid disease progression [37]. Importantly, overlap between antibody types can occur, and some patients may present with multiple autoantibodies or atypical profiles, further highlighting the complexity of SPSD [51]. Additionally, seronegative cases have been described, reinforcing the importance of clinical diagnosis even in the absence of detectable antibodies [52].

**Figure 2:**
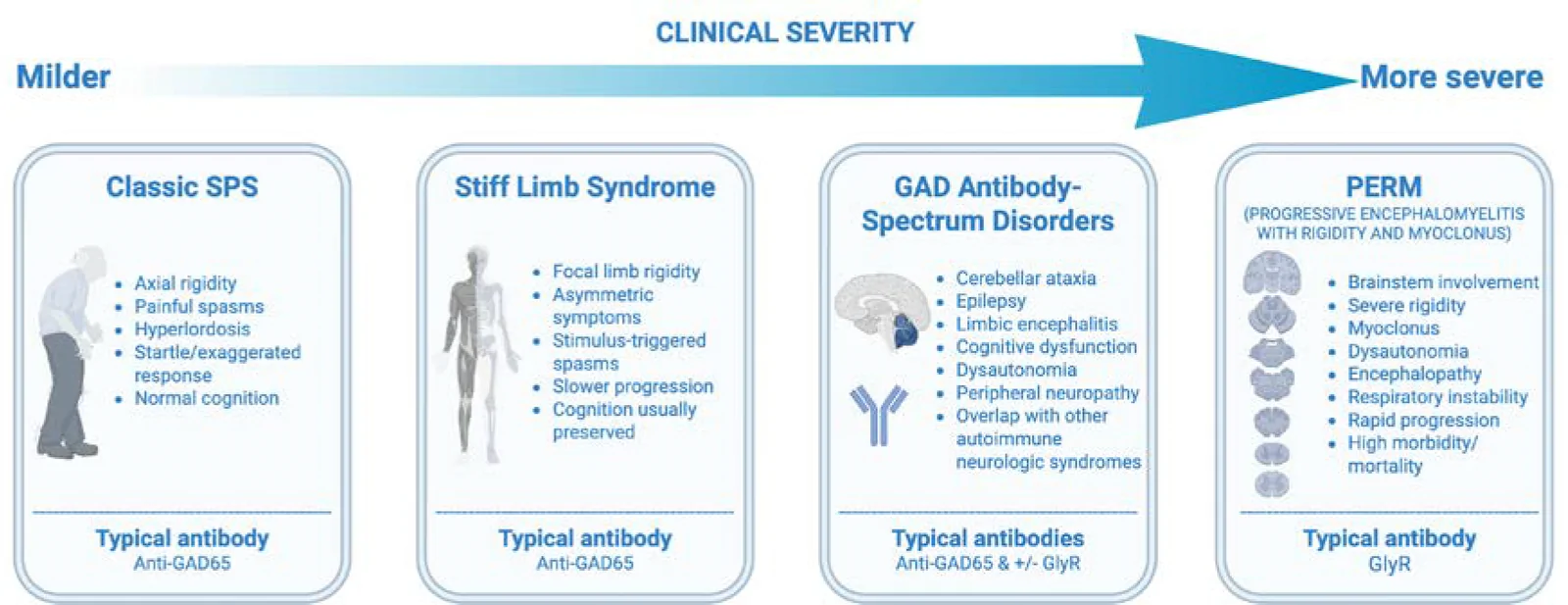
Clinical spectrum of Stiff Person Spectrum Disorder. GAD, glutamic acid decarboxylase; GlyR, glycine receptor.

SPSD encompasses a wide range of clinical manifestations beyond the classic features of rigidity and spasms (Figure 2). Patients may present with additional neurologic symptoms, including cerebellar dysfunction, seizures, cognitive impairment, and dysautonomia, depending on the regions of the central nervous system affected [8,9]. This heterogeneity contributes to significant diagnostic challenges, as SPSD may mimic other neurologic and psychiatric conditions, including epilepsy, functional movement disorders, and neurodegenerative diseases [15,53]. The variability in presentation also impacts disease course, with some patients experiencing a slowly progressive trajectory while others develop rapidly worsening, life-threatening complications.

Recognizing SPSD as a spectrum has important implications for both diagnosis and management. The identification of specific phenotypes and antibody profiles can help guide clinical evaluation, including the need for malignancy screening in paraneoplastic cases and more aggressive immunotherapy in severe variants. From a rehabilitation perspective, the degree of functional impairment varies widely across the spectrum. Patients with classic SPS may experience progressive limitations in mobility and activities of daily living, while those with severe forms such as PERM may develop profound disability requiring intensive multidisciplinary care. Understanding this variability is essential for tailoring rehabilitation strategies and optimizing functional outcomes.

Stiff person spectrum disorders represent a continuum of autoimmune neurologic conditions characterized by impaired inhibitory neurotransmission and diverse clinical manifestations. Recognition of this spectrum is critical for accurate diagnosis, appropriate risk stratification, and individualized management (Figure 2). As understanding of SPSD continues to evolve, integrating clinical phenotypes with immunologic profiles will be essential for advancing both therapeutic approaches and patient outcomes.

### CLINICAL PRESENTATION AND FUNCTIONAL IMPAIRMENT

Stiff person syndrome (SPS) is characterized by a progressive mixture of motor and non-motor symptoms resulting from impaired inhibitory neurotransmission. The classic clinical presentation includes axial rigidity, painful muscle spasms, and heightened sensitivity to external stimuli, which together contribute to significant functional impairment. Symptoms typically develop insidiously and progress over time, often leading to substantial disability if left untreated [11,12].

The hallmark feature of SPS is progressive stiffness involving the axial and proximal limb musculature, particularly affecting the paraspinal and abdominal muscles. This rigidity often leads to hyperlordosis and a characteristic stiff, en bloc gait. Patients may report difficulty with ambulation, impaired balance, and an increased risk of falls [13,31]. In many cases, stiffness fluctuates but gradually worsens, contributing to long-term functional decline. Superimposed on this baseline rigidity are episodic, often severe muscle spasms that can be triggered by external stimuli such as sudden noise, tactile stimulation, emotional stress, or voluntary movement. These spasms are frequently painful and may result in falls, injury, or transient functional incapacitation. The exaggerated startle response observed in SPS is a key clinical clue and reflects underlying disinhibition within central motor pathways [6,45].

Beyond motor symptoms, SPS is increasingly recognized as a disorder with significant neuropsychiatric manifestations. Anxiety, phobias, and hypervigilance are common and may develop before the onset of motor symptoms, sometimes leading to initial misdiagnosis as a primary psychiatric disorder [14,54]. Patients may develop task-specific fears, such as fear of walking in open spaces or crowded environments, due to the risk of stimulus-induced spasms. This overlap between neurologic and psychiatric features further complicates diagnosis and management.

SPS is also associated with a broad range of clinical phenotypes encompassed within stiff person spectrum disorders (SPSD). Variants such as stiff limb syndrome present with more localized rigidity, while progressive encephalomyelitis with rigidity and myoclonus (PERM) represents a more severe form characterized by brainstem involvement, autonomic instability, and encephalopathy [7,49]. Patients with PERM may exhibit additional features such as myoclonus, seizures, and dysautonomia, highlighting the heterogeneity of SPS presentations. In addition to neurologic symptoms, systemic manifestations and comorbidities are common. SPS frequently coexists with other autoimmune conditions, particularly type 1 diabetes and thyroid disease, and may be associated with paraneoplastic syndromes in certain cases [16,37]. Rare presentations include cognitive impairment, limbic encephalitis, and autonomic dysfunction, further expanding the recognized clinical spectrum [50,55].

Importantly, SPS is often misdiagnosed due to its rarity and overlapping features with other neurologic, psychiatric, and functional disorders. Patients may initially be diagnosed with conditions such as epilepsy, functional movement disorders, or musculoskeletal disorders before the correct diagnosis is ultimately established [15,53]. This diagnostic delay can result in prolonged morbidity and highlights the importance of clinical awareness. From a functional standpoint, SPS has a profound impact on mobility, independence, and quality of life. Patients frequently have trouble with activities of daily living, reduced endurance, and social isolation due to fear of triggering spasms. Functional impairment is often disproportionate to disease rarity, highlighting the need for early recognition and comprehensive management strategies [25,26].

Overall, the clinical presentation of SPS is heterogeneous, ranging from classic axial rigidity to severe multisystem involvement in SPS spectrum disorders. Recognition of both motor and non-motor features, as well as the functional consequences of the disease, is essential for timely diagnosis and effective multidisciplinary management.

### DIAGNOSTIC EVALUATION

The diagnosis of stiff person syndrome (SPS) is primarily clinical, supported by serologic and electrophysiologic findings. Due to the rarity of the condition and its overlap with psychiatric, functional, and other neurologic disorders, diagnosis is frequently delayed or initially missed. A high index of clinical suspicion is essential, particularly in patients presenting with progressive rigidity, stimulus-triggered spasms, and unexplained functional decline [14,19].

SPS is diagnosed based on a combination of characteristic clinical features, including axial and proximal muscle stiffness, painful spasms triggered by external stimuli, and exaggerated startle responses. Symptoms are typically chronic and progressive, often leading to significant impairment in mobility and activities of daily living over time [12,31]. Recognition of these hallmark features is critical, as early manifestations may be subtle or misattributed to anxiety, musculoskeletal conditions, or functional disorders [15,53].

Serologic evaluation plays a central role in supporting the diagnosis of SPS. The most detected antibodies are directed against glutamic acid decarboxylase (GAD65), which are present in most patients with classic SPS [6,9]. High titers of anti-GAD antibodies are more specific for SPS, and related neurologic disorders compared to lower titers seen in other autoimmune conditions. Additional antibodies associated with SPS spectrum disorders include glycine receptor (GlyR) antibodies, which are more commonly found in severe phenotypes such as progressive encephalomyelitis with rigidity and myoclonus (PERM), and amphiphysin antibodies, which are often associated with paraneoplastic SPS [7,10,37]. The identification of these antibodies can help guide both diagnosis and evaluation for underlying malignancy. However, it is important to note that serologic testing is not definitive. Some patients with clinically consistent SPS may have low or undetectable antibody levels, emphasizing the importance of clinical judgment in diagnosis [52].

Electromyography (EMG) is a key diagnostic tool in SPS and provides objective evidence of impaired inhibitory control. The characteristic finding is continuous motor unit activity at rest, reflecting involuntary and sustained muscle activation [6,45]. Additional findings may include impaired reciprocal inhibition and abnormal reflex responses, further supporting the diagnosis. Emerging neurophysiological techniques, including gait analysis and advanced EMG assessments, have demonstrated the potential to quantify disease severity and functional impairment, though their role in routine clinical practice remains limited [56,57].

Magnetic resonance imaging (MRI) of the brain and spinal cord is typically normal in patients with SPS and is primarily used to exclude alternative diagnoses. In some cases, particularly in SPS spectrum disorders such as PERM, imaging may reveal nonspecific abnormalities [11]. On the other hand, cerebrospinal fluid (CSF) analysis may demonstrate evidence of intrathecal antibody synthesis, including elevated anti-GAD antibody levels and oligoclonal bands, supporting a central autoimmune process [46]. However, CSF findings are variable and not required for diagnosis.

### DIFFERENTIAL DIAGNOSIS AND DIAGNOSTIC CHALLENGES

The differential diagnosis of SPS is broad and includes neurologic, psychiatric, and musculoskeletal conditions. Common considerations include Parkinson disease, dystonia, multiple sclerosis, tetanus, neuromyotonia, and ankylosing spondylitis. Functional movement disorders and psychogenic nonepileptic events are also frequently considered, particularly in patients with prominent psychiatric symptoms or atypical presentations [14,53]. Importantly, SPS may also mimic or be misdiagnosed as epilepsy due to stimulus-induced spasms and episodic symptoms [15]. Conversely, SPS-like presentations may arise from alternative conditions, including endocrine disorders such as hypopituitarism, further complicating diagnostic evaluation [58].

Despite advances in serologic and electrophysiologic testing, SPS remains a challenging diagnosis. The heterogeneity of clinical presentations, variability in antibody profiles, and overlap with other conditions contribute to frequent delays in diagnosis. Recent studies have proposed refined diagnostic criteria and core clinical features to improve diagnostic accuracy, though further validation is needed [59]. Ultimately, the diagnosis of SPS requires integration of clinical findings, supportive laboratory and electrophysiologic data, and exclusion of alternative diagnoses. Early recognition is critical, as timely initiation of therapy may improve functional outcomes and reduce disease progression.

### CLINICAL MANAGEMENT

The management of stiff person syndrome (SPS) requires a multimodal approach targeting both symptomatic relief and the underlying autoimmune process. Given the heterogeneity of SPS spectrum disorders, treatment strategies must be individualized based on disease severity, antibody profile, and functional impairment. Current therapies can be broadly categorized into symptomatic pharmacologic management, immunotherapy, and emerging or adjunctive interventions [60].

Symptomatic treatment aims to restore inhibitory neurotransmission and reduce muscle stiffness and spasms. Benzodiazepines, particularly diazepam, are considered first-line agents due to their potentiation of gamma-aminobutyric acid (GABA)-mediated inhibitory signaling. Baclofen, a GABA-B receptor agonist, is also commonly used and may be administered orally or intrathecally in refractory cases [12,61]. Additional agents such as gabapentin, levetiracetam, and tizanidine may be used as adjuncts to control spasticity and reduce symptom burden and pain [48]. In select cases, botulinum toxin injections have also been reported to improve focal rigidity and muscle spasms, particularly in cervical and paraspinal musculature [62]. For acute exacerbations or severe spasms, agents such as clonidine and ketamine have been described in case reports as potential options for crisis management, although evidence remains limited [63,64].

Immunotherapy represents the cornerstone of disease-modifying treatment in SPS, targeting the underlying autoimmune process. Intravenous immunoglobulin (IVIG) is the most well-established therapy, with randomized controlled trial data demonstrating significant improvement in stiffness, spasm frequency, and functional outcomes [44]. Subsequent studies have reinforced IVIG as first-line immunotherapy, particularly in patients with anti-GAD antibody-associated disease [65,66]. Corticosteroids and plasma exchange are also used in clinical practice, although evidence supporting their efficacy is not as strong. Rituximab, a monoclonal antibody targeting CD20-positive B cells, has emerged as an important option for refractory SPS. While randomized trial data have shown mixed results, systematic reviews suggest that rituximab may provide benefit in selected patients, particularly those with severe or treatment-resistant disease [22,67].

For patients with severe, refractory SPS, more aggressive immunomodulatory strategies are being further explored. Hematopoietic stem cell transplantation (HSCT) has shown promising results in case reports and small series, suggesting the potential for long-term disease control in selected patients [68,69]. More recently, chimeric antigen receptor T-cell (CAR-T) therapy has emerged as a novel approach targeting autoreactive B cells, with early reports demonstrating significant clinical improvement in otherwise refractory cases [21,23,24]. Other immunomodulatory approaches, including extracorporeal photopheresis, have been described as potential adjunctive therapies, though data remain limited [70]. Combination therapies, such as IVIG with plasma exchange or other immunosuppressive agents, may also be considered in complex cases [71].

Emerging evidence suggests that neuromodulation techniques may play a role in the management of refractory symptoms. Spinal cord stimulation has been reported to reduce rigidity and painful spasms in select patients, offering a potential option for symptom control when conventional therapies are insufficient [28,72]. Non-invasive neuromodulation approaches have also shown preliminary promise, although further study is needed [73].

### REHABILITATION MANAGEMENT

**Figure 3:**
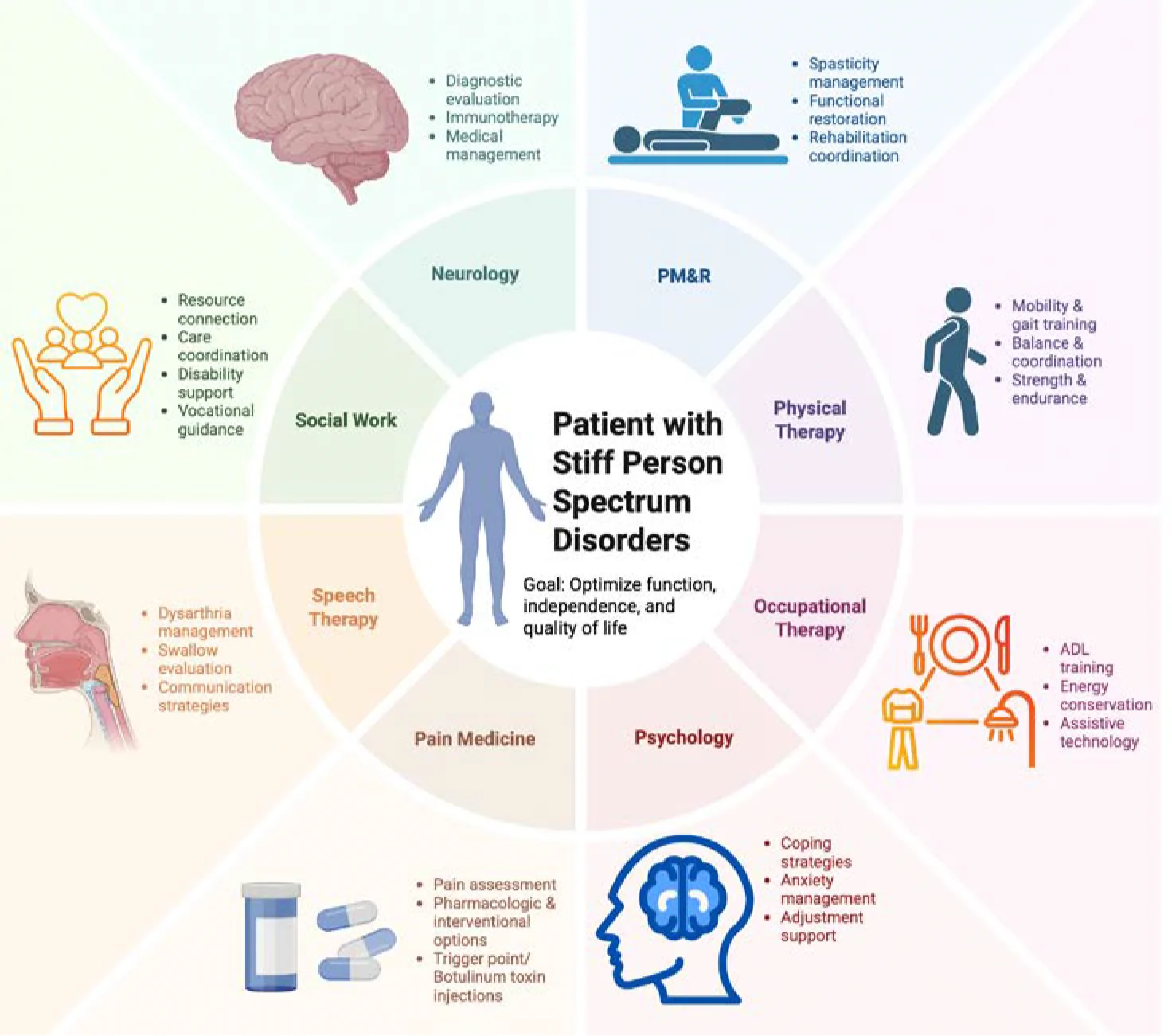
Comprehensive Physical Medicine and Rehabilitation (PM&R) management framework for Stiff Person Syndrome. ADL, activities of daily living.

Given the complex and disabling nature of stiff person syndrome (SPS), multidisciplinary care is essential (Figure 3). Rehabilitation interventions, including physical therapy, occupational therapy, and spasticity management, are critical components of treatment and can significantly improve functional outcomes [74–83]. Although evidence is largely limited to case reports and small series, rehabilitation consistently demonstrates benefits in mobility, independence, and quality of life [26,27]. Psychological support is also important, as anxiety and phobias are common and may exacerbate symptoms. In advanced disease, palliative care approaches may be necessary to address refractory symptoms and maintain quality of life [81,82,84]. SPS is associated with significant functional impairment due to progressive rigidity, painful spasms, and heightened stimulus sensitivity. These features can have a great impact on mobility, independence, and quality of life, making rehabilitation a critical component of comprehensive management. Despite this, rehabilitation strategies remain underrepresented in the literature, with most evidence derived from case reports and small series.

Patients with SPS commonly experience progressive limitations in mobility, including impaired gait, reduced balance, and increased risk of falls. Axial rigidity and hyperlordosis contribute to a stiff, en bloc gait pattern, while superimposed spasms may cause sudden loss of postural control. These impairments often lead to difficulty performing activities of daily living (ADLs), reduced endurance, and social withdrawal [25,26]. Recent efforts to quantify functional impairment have highlighted the substantial burden of disease. Functional outcome studies and emerging tools such as the SPS-specific activities of daily living (SPS-ADL) score demonstrate that SPS can result in significant disability, even in patients receiving medical therapy [25,85]. These findings underscore the importance of incorporating functional assessments into routine clinical care.

Physical therapy plays a central role in the management of SPS, with interventions aimed at improving flexibility, posture, and functional mobility. Common strategies include stretching programs to reduce muscle stiffness, gait training to improve ambulation, and balance exercises to reduce fall risk. Occupational therapy interventions focus on optimizing independence in daily activities and adapting the environment to minimize triggers for spasms. Strategies may include the use of assistive devices, modification of home and work environments, and training in compensatory techniques for ADLs. Addressing functional limitations early can help prevent deconditioning and reduce the risk of long-term disability. Case-based evidence suggests that both inpatient and outpatient rehabilitation programs can lead to meaningful improvements in mobility and independence [26,27,86]. Given the risk of stimulus-induced spasms, therapy programs should be individualized and progressed cautiously. Gradual exposure to movement and environmental stimuli may help patients build tolerance and reduce fear-avoidant behaviors. Education on energy conservation and pacing strategies is also essential, particularly in patients with severe disease (Figure 3).

Spasticity and pain are major contributors to functional impairment in SPS and often require a multimodal management approach. In addition to systemic medications such as baclofen and benzodiazepines, targeted interventions may be beneficial. Botulinum toxin injections have been reported to reduce focal muscle rigidity and improve range of motion, particularly in the cervical and paraspinal musculature [62]. Neuromodulation techniques are an emerging area of interest in SPS management. Spinal cord stimulation has demonstrated potential in reducing rigidity and painful spasms in refractory cases, suggesting a role for interventional pain management in selected patients [28,72]. Non-invasive neuromodulation approaches have also shown early promise, although further research is needed.

Neuropsychiatric symptoms, including anxiety, phobias, and hypervigilance, are common in SPS and can also significantly impact functional outcomes. These symptoms often exacerbate motor manifestations, as stress and emotional stimuli may trigger spasms. Cognitive behavioral therapy and other psychological interventions may help patients manage anxiety and improve participation in rehabilitation [54].

Given the complexity of SPS, a multidisciplinary approach is essential for optimal care. Collaboration between neurology, physical medicine and rehabilitation, physical and occupational therapy, and mental health professionals is critical to address both the neurologic and functional aspects of the disease. Despite consistent reports of functional improvement with rehabilitation, high-quality evidence remains limited. Most available data are derived from case reports and small observational studies, with a lack of standardized outcome measures and controlled trials. Future research is needed to establish evidence-based rehabilitation protocols and to better define the role of emerging interventions, including neuromodulation and multidisciplinary care models.

### FUNCTIONAL OUTCOMES AND QUALITY OF LIFE

Stiff person syndrome (SPS) is associated with a substantial functional burden that extends beyond its core neurologic manifestations. Progressive muscle rigidity, painful spasms, and heightened sensitivity to external stimuli contribute to significant impairments in mobility, activities of daily living (ADLs), and overall quality of life. Despite advances in immunotherapy and symptomatic management, many patients experience persistent functional limitations that require long-term multidisciplinary care.

Functional impairment in SPS is driven by a combination of axial rigidity, impaired motor control, and unpredictable, stimulus-triggered spasms that contribute to abnormal gait patterns, decreased balance, and an increased risk of falls. Patients frequently develop a stiff, en bloc gait and may avoid movement due to fear of triggering spasms, further worsening functional decline [13,25]. Recent studies have demonstrated that SPS is associated with significant disability across multiple domains, including mobility, self-care, and participation in social and occupational activities. Functional outcome analyses also suggest that even patients receiving medical therapy continue to experience measurable impairments, highlighting the chronic and disabling nature of the disease [25]. In more severe cases, particularly within stiff person spectrum disorders, patients may become dependent on assistive devices or require caregiver support for basic activities.

Impairments in ADLs are a central feature of SPS and reflect both physical and psychosocial limitations. Patients commonly report difficulty with tasks such as walking, transferring, dressing, and bathing. The episodic nature of spasms makes functional performance more complicated, as patients may experience sudden, unpredictable limitations. Efforts to quantify functional impact have led to the development of disease-specific tools, such as the SPS activities of daily living (SPS-ADL) score, which provides a structured assessment of functional impairment. In addition, objective measures such as gait analysis and electromyography-based assessments have been explored to quantify motor dysfunction and monitor disease progression [56,85]. However, standardized outcome measures remain limited, representing an important area for future research.

The impact of SPS on quality of life is multifaceted, encompassing physical, emotional, and social domains. Chronic pain, mobility limitations, and fear of spasms can lead to reduced participation in daily activities and social isolation. Anxiety, phobias, and hypervigilance are common and may significantly exacerbate functional impairment [54]. Patients often develop anticipatory anxiety related to environmental triggers, such as walking in crowded spaces or navigating uneven terrain. This fear-avoidant behavior can further restrict mobility and independence, creating a cycle of deconditioning and functional decline. In severe cases, these psychosocial factors may contribute as much to disability as the underlying motor symptoms.

Functional outcomes in SPS vary widely depending on disease severity and phenotype. Patients with classic SPS may experience gradual functional decline over time, whereas those with more severe variants, such as progressive encephalomyelitis with rigidity and myoclonus (PERM), may develop rapid and profound disability, including autonomic dysfunction and cognitive impairment [49]. Long-term outcome data suggest that SPS often follows a chronic course with variable progression, emphasizing the importance of early intervention and ongoing management [13,87]. The heterogeneity of SPS spectrum disorders makes prognosis prediction increasingly difficult and underscores the need for individualized care plans.

Improving functional outcomes in SPS requires a comprehensive, multidisciplinary approach that integrates medical management with rehabilitation and psychosocial support. Physical and occupational therapy interventions can help address mobility limitations, reduce fall risk, and improve independence in ADLs. Spasticity management, pain control, and neuromodulation may further enhance functional capacity in selected patients [27,28]. Psychological interventions, including cognitive behavioral therapy, may be helpful in addressing anxiety and fear-avoidant behaviors, thereby improving participation in rehabilitation and daily activities. In advanced cases, palliative care approaches may be necessary to optimize quality of life and manage refractory symptoms [84].

SPS is associated with significant and often underrecognized functional impairment and reduced quality of life. While advances in immunotherapy have improved disease control, functional limitations frequently persist and require targeted rehabilitation and multidisciplinary management. Greater emphasis on functional outcomes, standardized assessment tools, and patient-centered care is essential for improving the overall well-being of individuals living with SPS.

### FUTURE DIRECTIONS

Despite significant advances in the understanding of stiff person syndrome (SPS), substantial gaps remain in diagnosis, treatment, and long-term management. Emerging research is increasingly focused on improving disease characterization, identifying reliable biomarkers, and developing targeted therapies to address the underlying autoimmune mechanisms.

One of the most important areas of future research is the identification of biomarkers that can improve diagnostic accuracy, predict disease course, and guide treatment selection. Although anti-glutamic acid decarboxylase (GAD) antibodies remain the most used diagnostic marker, variability in antibody titers and the presence of seronegative cases limit their use as a definitive diagnostic tool. Recent studies have identified potential immunogenetic associations, including lymphocytic gene variants and immune regulatory pathways, which may contribute to disease susceptibility and heterogeneity [35]. In addition, emerging data on glycine receptor (GlyR) antibodies and other immune targets suggest that SPS may include multiple distinct immunologic subtypes. A more specific classification of SPS spectrum disorders based on antibody profiles and molecular mechanisms could facilitate a precision medicine approach, allowing for more individualized treatment strategies [8,9].

While intravenous immunoglobulin (IVIG) remains the most evidence-supported therapy, treatment responses are variable and many patients experience refractory disease. Newer immunotherapies targeting specific components of the immune system are an area of active investigation. B-cell-depleting therapies such as rituximab have shown promise in selected patients, although further studies are needed to identify predictors of response [22]. More recently, cellular therapies such as chimeric antigen receptor T-cell (CAR-T) therapy have demonstrated encouraging results in severe, treatment-resistant SPS, offering the potential for long-term disease remission [23,24]. Similarly, hematopoietic stem cell transplantation (HSCT) has emerged as a potential option for refractory cases, although its role remains to be clearly defined [68]. Additional immunomodulatory strategies, including extracorporeal photopheresis and combination therapies, are also being explored [70].

Advances in neurophysiological testing and imaging techniques may enhance diagnostic accuracy and provide objective measures of disease severity. Electrophysiologic studies, including electromyography and advanced gait analysis, have shown potential in quantifying motor dysfunction and monitoring treatment response [45,56]. Recent efforts to define core diagnostic features and develop standardized criteria may also help reduce diagnostic delays and improve early recognition of SPS [59].

Furthermore, the development of disease-specific functional assessment tools, such as the SPS-ADL score, represents an important step toward standardized outcome measurement in both clinical practice and research [85].

Although rehabilitation is becoming more recognized as a critical component of SPS management, high-quality evidence remains limited. Future studies are needed to establish standardized rehabilitation protocols, evaluate the efficacy of specific interventions, and determine optimal timing and intensity of therapy. The early integration of physical therapy, occupational therapy, psychological support, and interventional pain management into a cohesive care model may significantly improve functional outcomes and quality of life. Emerging modalities such as neuromodulation, including spinal cord stimulation and non-invasive techniques, warrant further investigation as potential adjunctive therapies for symptom control [28,73]. Additionally, the role of complementary therapies, such as acupuncture, remains an area for future exploration [88].

SPS is a highly heterogeneous condition with variable clinical presentations, antibody profiles, and treatment responses. Long-term, prospective studies are needed to better understand disease progression, identify prognostic factors, and evaluate the durability of treatment effects [13,87]. Greater emphasis on patient-reported outcomes and functional measures will also be important in capturing the full impact of the disease. Finally, increased awareness and earlier diagnosis remain critical goals. Educational initiatives aimed at improving recognition of SPS among healthcare providers may help reduce diagnostic delays and improve patient outcomes.

### CONCLUSION

Stiff person syndrome (SPS) is a rare but highly disabling autoimmune neurologic disorder characterized by impaired inhibitory neurotransmission, progressive rigidity, and stimulus-triggered muscle spasms. Advances in the understanding of its immunopathogenesis, particularly the role of anti-GAD and glycine receptor antibodies, have redefined SPS as part of a broader spectrum of autoimmune neurologic disorders with diverse clinical manifestations. Despite these advances, SPS remains a diagnostic challenge due to its rarity, heterogeneous presentation, and frequent overlap with psychiatric, functional, and other neurologic conditions. Early recognition is critical, as timely diagnosis and initiation of therapy can significantly improve patient outcomes and reduce long-term disability. Current treatment strategies emphasize both symptomatic management and immunotherapy, with intravenous immunoglobulin representing the most evidence-supported intervention, while emerging therapies such as rituximab, hematopoietic stem cell transplantation, and CAR-T therapy offer promise for refractory disease.

Importantly, SPS carries a substantial functional burden that extends beyond neurologic symptoms, affecting mobility, independence, and quality of life. Rehabilitation interventions, including physical therapy, spasticity management, and neuromodulation, play a vital role in improving functional outcomes, yet remain underrepresented in the literature. A multidisciplinary approach that integrates neurologic, rehabilitative, and psychosocial care is essential for comprehensive management. Future efforts should focus on improving diagnostic accuracy through biomarker development, advancing targeted immunotherapies, and establishing evidence-based rehabilitation strategies. Greater emphasis on functional outcomes and patient-centered care will be critical in addressing the full impact of the disease. In summary, SPS represents a complex and evolving disorder at the intersection of neuroimmunology and rehabilitation medicine. A comprehensive approach that combines advances in immunotherapy with a strong focus on functional recovery offers the greatest potential to improve outcomes for affected patients.

## KEY TAKEAWAYS

- Stiff person syndrome (SPS) is a rare autoimmune neurologic disorder driven by impaired inhibitory neurotransmission and motor neuron hyperexcitability.
- SPS exists within a broader stiff person spectrum disorder (SPSD) encompassing phenotypes ranging from focal rigidity to life-threatening encephalomyelitis (PERM).
- The disease is strongly associated with autoantibodies (anti-GAD65, glycine receptor, amphiphysin) that correlate with clinical phenotype and severity.
- Diagnosis remains challenging and is often delayed due to clinical heterogeneity and frequent misdiagnosis as psychiatric, functional, or epileptic disorders.
- SPS is closely linked to systemic autoimmunity, particularly type 1 diabetes and thyroid disease, and may also present as a paraneoplastic syndrome.
- Intravenous immunoglobulin (IVIG) is the most evidence-supported therapy, while treatments such as rituximab and other immunotherapies show variable benefit.
- Emerging therapies, including CAR-T, hematopoietic stem cell transplantation, and neuromodulation, offer promising options for refractory disease.
- SPS imposes a significant functional burden, with impairments in mobility, balance, and activities of daily living that persist despite medical treatment.
- Rehabilitation plays a critical but underrecognized role, with evidence suggesting improvements in function, independence, and quality of life.
- Future directions should prioritize biomarker development, standardized outcome measures, and multidisciplinary care models to optimize diagnosis and long-term management.
